# COVID-19 pandemic and the changing views of mobility: the case of Nepal–Malaysia migration corridor

**DOI:** 10.1186/s40878-022-00320-1

**Published:** 2022-11-01

**Authors:** Andika Wahab, Mashitah Hamidi

**Affiliations:** 1 Institute of Malaysian & International Studies (IKMAS), National University of Malaysia (UKM), Bangi, Selangor, Malaysia; 2 Department of Social Justice and Administration, University of Malaya (UM), Kuala Lumpur, Malaysia

**Keywords:** COVID-19, Mobility, Migrant workers, Aspiration-ability model

## Abstract

For decades, Malaysia has been heavily dependent on unskilled and temporarily contracted migrant workers to fulfil labour gaps in the country. While Malaysia’s economy continues to rely on migrant workers, the COVID-19 pandemic has further aggravated their precarious working and living conditions. In-depth interviews with Nepali migrant workers and community leaders in Malaysia and Nepal in 2021 revealed the incidence of labour rights violations, compounded by the lack of access to justice and effective remedies. Besides, workers are allegedly no longer benefiting from the competitive wages, subsequently limiting the value of their remittance to Nepal. We argue that these incidents serve as the drivers of the changing views of mobility, eventually influencing the emigration environment in which the social construction of migration exists in Nepal. This study examines the migratory realities in the Nepal–Malaysia migration corridor during the pandemic, subsequently contributing to current debate on the aspiration–ability model as a class of research.

## Introduction

In countries that host a significant number of international migrants, the COVID-19 pandemic’s epicentre is often located in the migrant population. In early May 2020, when active COVID-19 cases among the population of Kuwait, United Arab Emirates (UAE), and Bahrain ran in the single digits, thousands of migrant workers were still ill with COVID-19 and under quarantine (Geneva Solution, 2020). Similarly, in Singapore, as of 6 May 2020, about 88 per cent (or 17,758) of the total 20,198 cases identified as positive for COVID-19 were among migrant workers living in employer-sponsored dormitories (College of Family Physicians Singapore, 2020). Existing literature has linked the rapid transmission of COVID-19 with poor working and living conditions facing migrant workers in destination countries, for example, overcrowded dormitories and unsanitary conditions (Wahab, 2020). The pandemic is seen as an emerging source of insecurities that challenge migrants, their livelihoods and broader migration governance (Murzakulova et al., 2021).

Prior to the COVID-19 outbreak, migrant workers in major destination countries such as Kuwait, UAE, South Korea, Malaysia, and Singapore (just to mention a few) were already facing poor access to healthcare due to a lack of inclusive social protection, compounded by administrative hurdles and language barriers. This situation became aggravated during the outbreak as migrant workers are less likely to be able to practice physical distancing due to overcrowded living conditions and workspaces. Also, as migrant workers live in temporary communities separate from the general population, they are often ‘forgotten’ and not considered in national actions and policies during such crises as the COVID-19 outbreak.

On 11 March 2020, the World Health Organization (WHO) declared COVID-19 as a pandemic. By the end of March 2022, COVID-19 had spread to nearly 200 countries and territories, with over 400 million people having been infected by the virus and its variants globally, and close to 7 million deaths. Malaysia confirmed its first COVID-19 case on 25 January 2020. Malaysia took early preventive action by implementing the Movement Control Order (MCO) on 18 March 2020, followed by different versions and phases of MCOs, until the recent time in September 2021.

The COVID-19 outbreak and continuous imposition of stricter MCOs (i.e., lockdown) between 2020 and late 2021 raise a question as to how migrant workers in Malaysia are impacted. In this article, we focus on Nepali migrant workers, with the aim to examine their everyday employment and life experiences during the course of the pandemic and to ascertain whether (or not) their present situations influence future migration from Nepal (to Malaysia). In this article, we leverage the aspiration-ability model, with two main research inquiries in mind, namely, (i) how the present situations (during the COVID-19 outbreak) facing Nepali workers in Malaysia change their views of mobility to Malaysia? and, (ii) how do the changing views of mobility among the existing Nepali workers in Malaysia influence or shape (or not) the emigration environment in which social construction of migration exists in Nepal?

This study relies on primary data, derived from key informant interviews with three groups of informants, namely, (i) the Nepali workers in Malaysia, (ii) Nepali community leaders in Malaysia; and (iii) Nepali community leaders in Nepal—all of which were conducted between 15 May 2021 and 30 September 2021. The first group of informants involved 28 Nepali workers who were working at the time of the interview, in Klang Valley, Penang and Johor in Malaysia. The interview sessions were conducted in a hybrid mode, namely, face-to-face, and remote interviews. The interview sessions used semi-structured interview questions, aiming to understand detailed accounts of their migration experiences, and working conditions in Malaysia. As for the second group of informants, it involved separate interview sessions with 13 Nepali community leaders in Malaysia. The third group of informants involved five other Nepali community leaders in Nepal, who were interviewed fully virtually, in several separate sessions. The interview with Nepali community leaders in Malaysia and Nepal aimed to understand the migration policy development and implementation in Nepal and to solicit migration narratives in Nepal during and post COVID-19 period.

The next section illustrates the current Malaysian labour market sentiment that is highly dependent on the migrant workforce, followed by a review of the migration aspiration and ability model, and a critical reflection of migration governance in the Nepal–Malaysia migration corridor. We then present the key drivers of the changing views of mobility among the key informants. Thereafter, we discuss the intersection between the changing views of mobility and the migration aspiration and mobility model, and finally, we present our conclusion.

## Malaysian labour market and its reliance on the migrant workforce

For the past decades and so, Malaysia had successfully diversified its economy from agriculture and commodity-based alone, to include manufacturing and service sectors, making it one of the leading exporters of products globally (e.g., electrical appliances and components). Such economic diversification paved the way for job creation and income growth (World Bank, 2022), concurrently attracting more foreign investments and development projects implemented in the country (Solomon et al., 2015). Malaysia is also committed to making a transition from an upper-middle income to a high-income economy by 2024 (see Devadas et al., 2020; World Bank, 2022), though some economic sectors including plantation, agriculture and manufacturing are still heavily dependent on manual, unskilled and low-paid workers (see Hamzah et al., 2020; Arisman & Jaya, 2020; Shahiri et al., 2021).

Accompanying this labour market situation, existing studies also indicate that local workers are not keen to fill these manual, unskilled and low-paid jobs, and associate such work as “dirty, demeaning and dangerous” (Katmon et al., 2020; Mohd Fateh et al., 2022). At the same time, employers tend to hire migrant workers, as opposed to local workers (Ismail, 2015), for a variety of reasons, including the likelihood of working for a longer period (e.g., working up to 10 years for the same employer), willingness to work in hardship conditions, readiness to work in longer hours of work with relatively low wages—cumulatively offers a sense of workforce stability.

Given the current labour market sentiment and high level of dependency on the migrant workforce, Malaysia becomes one of the major destination countries for international migrant workers in the region. Existing estimates indicate as few as 2 million up to 5.5 million migrant workers live in Malaysia, consisting of documented and undocumented migrant workers (Lee et al. 2018). While the exact number of undocumented migrant workers cannot be accurately determined, the Malaysian authorities keep a record of the number of documented migrant workers—locally termed as migrant workers with visitor’s pass temporary employment (VPTE). As of early 2019, there were about 2 million migrant workers who were given VPTE in Malaysia, the majority of whom were workers from Indonesia, Bangladesh, Nepal, India, and Myanmar—working in five formal sectors (i.e., construction, manufacturing, services, plantations, and agriculture) and domestic service (Ministry of Human Resources, 2019) (Table 1).

**Table 1:** Number of Migrant Workers with Visit Pass Temporary Employment (VPTE) in Malaysia, 2019

Nationality	Sectors in Malaysia						Total
	Domestic Worker	Construction	Manufacturing	Services	Plantations	Agriculture	
Indonesia	90,718	162,421	136,394	43,325	205,177	75,890	713,925
Bangladesh	122	197,796	206,843	85,350	34,657	19,884	544,652
Nepal	60	7,927	251,503	73,892	3,045	9,886	346,313
India	1,059	11,033	2,902	51,142	28,603	26,239	120,978
Myanmar	66	11,704	79,186	16,151	906	3,789	111,802
*Others	35,144	44,121	28,188	36,557	10,106	23,974	178,090
Total	127,169	435,002	705,016	306,417	282,494	159,662	2,015,760

*Include such countries as Pakistan, the Philippines, Vietnam, China, Thailand, Sri Lanka, Cambodia and Laos

Source: Ministry of Human Resources (MOHR), 2019

Though the Malaysian economy is heavily dependent on the migrant workforce, existing studies, prior to the COVID-19 outbreak, had already highlighted a range of labour rights violations against migrant workers, committed by unscrupulous employers and intermediaries along the recruitment and labour migration chains. These include, amongst others, the unlawful withholding of passports and other identity documents (Fair Labor Association & Consumer Good Forum, 2018; Earthworm Foundation, 2019; Wahab, 2019); non-payment of wages; and other practices that restrict workers’ freedom of movement, their right to association and collective bargaining (Asia Monitor Resource Centre, 2019; Kanapathy, 2008; Kaur, 2010; Mei Wei & Yazdanifard, 2015). Such violations are further compounded by the lack of state protection and effective remedies to resolve labour and protection issues facing migrant workers (Wahab, 2020). Concurrently, there had been persistent stereotypes against migrant workers in the country, perceiving them as disposable workers (Ormond & Nah, 2020), and being disease vectors and financial burdens to the state (Juliawan, 2018; Ormond & Nah, 2020; Sok, 2019).

It is also widely acknowledged that migrant workers (including Nepali workers) deployed to Malaysia were initially recruited through a complex recruitment process in the country of origin—involving layers of labour agents, brokers, middle persons, and social networks (Kanapathy, 2008). The complex nature of their recruitment process has many negative consequences, one of which is the multiplication of recruitment costs borne by the workers (Kanapathy, 2008; Wahab, 2019). In many instances, irresponsible practices and manipulation during the recruitment process led to the high cost of migration imposed by agents, brokers, and recruitment agencies towards migrant workers (Kanapathy, 2008), and that subsequently situates the workers at risk of many forms of labour exploitation, including working in debt bondage situations in Malaysia.

## Reviewing migration aspiration-ability model

The COVID-19 pandemic has further exposed and aggravated the precarious working and living conditions of migrant workers in Malaysia (see Geneva Solution, 2020; Theng et al., 2020; Wahab, 2020; Kalra, 2021). In this article, we first argue that such precarious conditions serve as the drivers of the changing views of mobility, particularly among Nepali workers in Malaysia. This then raises two main research inquiries, (i) how these changing views of mobility intersect or shape the emigration environment back in Nepal, and (ii) what this implies to the future migration landscape in the Nepal–Malaysia migration corridor. It is important to highlight that this study does not intend to forecast the potential trend of labour migration in the Nepal–Malaysia migration corridor. Instead, we aim to examine the narratives and the likely impacts of the changing views of mobility, including aspirations, wishes, desires, and barriers for future labour mobility in the Nepal–Malaysia migration corridor.

Understanding and examining these narratives are certainly a challenging exercise. This cannot be accomplished academically persuasive without linking them with the existing body of knowledge. We locate these two main research inquiries in the context of migration aspiration and ability model—a framework that has been initially introduced by Carling (2002) about two decades ago, but constantly debated, adapted, and refined until recent years. This is the most logical and practical model to be used in the context of this study. According to Carling (2002), without resorting to overly structural explanations—migration is simply the combined results of two factors: the aspiration to migrate, and the ability to migrate. Specifically, Carling (2002: 2) defines migration aspiration as “… *a conviction that migration is preferable to non-migration: it can vary in degree and in the balance between choice and coercion*”. Important to note that not everyone who is aspired to migrate can do so. Carling (2002) refers to this specific group of people as involuntary non-migrants. Another group of people consists of those who stay because of a belief that non-migration is preferable to migration. Carling (2002) refers them as voluntary non-migrants.

The migration aspiration and ability model appears to be a logical and an easy to grasp framework. However, factors that determine migration aspiration and migration ability are more complex and multifaceted in nature. Carling (2002: 2) notes that “… *the aspiration to migrate emerges within a particular macro-level emigration environment, encompassing the social, economic and political context in which particular social constructions of migration exist*”. Carling (2002) further stresses that individual characteristics play an important role to determine patterns of who aspires to leave and who aspires to stay. Similarly, the ability to migrate is also conditioned by the macro-level of obstacles and opportunities (Carling, 2002), including immigration controls, family reunification, and the restricted characteristics of social and global mobility (see Vigh, 2009).

A more recent study co-authored by Duvell and Preiss (2022) claims that migration infrastructure, which consists of five complex and dynamic interplay between nature, technology, structure, agency, and knowledge—plays an important role to overcome these macro-level obstacles, including physical barriers and restrictive migration regulations and mobility restrictions. Specifically, there have been several scholarly attempts to explain the emergence of digital migration facilities to enable migration in times of growing mobility restrictions. Preiss (2022), for instance, argues that digitalization has fundamentally shaped the way people migrate, for example, using mobile phones for navigation, communicating with smugglers and families back home, using mobile money transfer, and in the case of irregular migration—mobile phones serve as lifelines between boats and rescue vessels.

Later in 2018, Carling together with Schewel (2018) revisited and reworked Carling’s aspiration and ability model introduced in 2002 and stressed that the model still has its own theoretical and methodological questions. To address these questions, Carling and Schewel (2018) introduced a two-step approach to better understand migration as a class of research: the evaluation of migration as a potential course of action, and the realisation of actual mobility or immobility. This two-step approach considers thoughts (e.g., perceptions) and feelings (e.g., wishes and desires) important to determine migration outcomes. They also note that migration aspiration and ability are two universally meaningful concepts to better understand the diverse and contemporary migratory realities.

In the past decades, scholars have introduced several important concepts to help understand and reflect on international mobility, especially in the context of growing mobility restrictions and obstacles. These include migration industries, migration facilities and humanitarian migration (see Duvell & Preiss, 2022; Preiss, 2022). In this article, we refer to and leverage the aspiration and ability model to better understand the diverse migratory realities in the Nepal–Malaysia migration corridor. However, our preliminary observations found that the model is yet to adequately address two important aspects: first, the digitalization process that changes many different aspects of mobility, and second, its readiness to explain migratory realities in times of great uncertainties such as the global COVID-19 pandemic. Here lies our scholarly contribution to the evolution of the migration aspiration and ability model.

## Research context: Migratin governance in the Nepal–Malaysia corridor

The presence of Nepali migrant workers in Malaysia’s domestic labour market is highly significant, making it (i.e., Nepal) among the top three source countries supplying migrant workers in Malaysia: the other two are Indonesia and Bangladesh. From years 2000 to 2020, they were nearly two million labour permits have been issued by the Government of Nepal to Nepali citizens to work in Malaysia (MoLESS, 2020). Foreign employment among Nepali workers abroad contributes significantly to the country’s development: nearly 50 per cent of Nepali households rely on financial help from relatives abroad (Kunwar, 2020). Malaysia is among the top-five destination countries for Nepali migrant workers: the other four countries include Saudi Arabia, Qatar, United Arab Emirates (UAE) and Kuwait (ILO, 2017). Given the significant cross-border labour migration in the Nepal–Malaysia migration corridor, the movement of Nepali workers into Malaysia’s labour market is governed by a complex interplay between regulatory agencies, domestic legislations and bilateral agreements, and privatized migration facilities that exist in both countries.

In Nepal, foreign employment among Nepali workers abroad is governed by the Ministry of Labour, Employment and Social Security (MoLESS). MoLESS, The Department of Foreign Employment (DOFE) under MoLESS is entrusted to formulate and implement a range of national and employment policies governing foreign employment of Nepali workers abroad. These include the Foreign Employment Act (2007), Foreign Employment Rules (2008) and Foreign Employment Policy (2012). The FER (2008), for instance, governs the creation of welfare funds for Nepali workers abroad, insurance coverage, pre-departure orientation and training, compensation, rescue, and repatriation. The FER (2008) also specifies the establishment of a migration monitoring facility located at the Tribhuvan International Airport (Nepal), and a foreign employment tribunal to address grievances received from Nepali workers abroad. The FEP (2012) was formulated to support the realization of the FER (2008) and to ensure effective management of foreign employment among Nepali workers abroad. To facilitate foreign employment among Nepali workers abroad, the Nepali Government has established a Foreign Employment Information Management System (FEIMS): a system that manages and regulates the labour migration among Nepali workers, including those in Malaysia.

As of 2020, the Government of Nepal has signed bilateral agreements with nine destination countries such as Qatar, UAE, the Republic of South Korea, Bahrain, Japan, Israel, Jordan, Malaysia, and Mauritius (ILO, 2017). The detailed contents of such agreements are varied, depending on specific objectives and expected outcomes mutually agreed upon between Nepal and the specific destination country such as Malaysia. The Government of Nepal has been consistently demanding the governments of destination countries to ensure such standards as employers pay principle, standard employment contract, access to justice and equality of treatment—are contained and elaborated in each bilateral agreement signed by both parties (Kunwar, 2020).

The private sector plays an important role in the recruitment of Nepali workers for foreign employment. To put into context, about 90 per cent of Nepali workers working abroad are recruited by private recruitment companies, before sent to destination countries such as Malaysia (MoLESS, 2020). Private companies are also entrusted to provide other foreign employment services to Nepali workers such as pre-employment training and orientation, medical test, legal and administrative support, and insurance and financial assistance. As of the end of 2020, there were a total of 854 recruitment agencies authorized by the Nepali Government to conduct recruitment of Nepali workers for foreign employment; 148 orientation centres to conduct pre-employment training; 226 medical centres accredited to perform the medical test; and 23 financial institutions and 14 insurance companies entrusted to provide financial supports to Nepali workers (MoLESS, 2020).

Besides these authorized private companies, recruitment of Nepali workers for foreign employment is often conducted, by way of a common practice, in partnership with individual subagents or brokers. These individual subagents or brokers act as intermediaries between the authorized recruitment agencies those that are often located in the capital city of Nepal, and the prospective Nepali workers who are mostly originated from remote cities and rural villages across the country. In March 2019, the Government of Nepal amended the Foreign Employment Act (2007) to prohibit the use of individual subagents or brokers as intermediaries in the recruitment of Nepali workers for foreign employment. Instead, the amended FEA (2007) allowed the recruitment agencies to establish branch offices outside the capital city to facilitate the recruitment process in rural and remote cities in Nepal (Mandal, 2020). In 2019, the Government of Nepal issued strict protocols to further strengthen the monitoring mechanisms against authorized recruitment agencies across the country. Despite these efforts, public reports continue to grow concerning unethical recruitment practices, frauds and deception involving tens of thousands of Nepali workers intending to work abroad (see MyRepublica, 2021; Nepal Minute, 2022).

Whilst, on the Malaysian side, the promulgation of the Policy on the Recruitment of Foreign Workers, followed by the introduction of a levy system in 1991 constituted a turning point in Malaysia’s governance system administering international labour migration into the country (Kaur, 2014). Migrant workers' policy in Malaysia has been, for at least these two decades and so, formulated and implemented as an ‘interim solution’ to reduce the hiring of undocumented migrant workers but also at the same time to reduce dependency on the foreign workforce. Statistically, there has been a steady increase in the number of work permits issued by the Government of Malaysia to international migrant workers from around 800,000 in 2000 to two million in 2019 (Ministry of Human Resources, 2019).

In Malaysia, the hiring of international migrant workers are governed by two key ministries: the Ministry of Human Resources (MOHR), and the Ministry of Home Affairs (MOHA). Concerning regulatory frameworks, like Nepal, there is a myriad of legislative frameworks and administrative requirements governing the recruitment and hiring of international migrant workers in Malaysia. The main legislation is the Employment Act (1955) which defines a ‘foreign employee’ (i.e., migrant worker) as ‘an employee who is not a citizen of Malaysia. The Act requires employers to furnish information on migrant workers (to be hired) and prohibits the termination of a local worker in order to employ a migrant worker. This policy approach was designed by the Government of Malaysia as a safeguard to protect local workers, and at the same time, to reduce reliance on the foreign workforce in the labour market.

Another important piece of legislation that deals with the recruitment and hiring of migrant workers is the Immigration Act 1959/63 (Act 155). The act regulates the entry of non-citizens of Malaysia, including migrant workers through the issuance of VPTE. Other relevant legislation and policies concerning the recruitment and hiring of migrant workers include, (i) the Anti-Trafficking in Persons and Anti-Smuggling of Migrants (ATIPSOM) Act 2007; (ii) the Employees’ Minimum Standards of Housing, Accommodations and Amenities Act 1990; (iii) Industrial Relations Act (IRA) 1967; (iv) Trade Union Act 1959; and (v) Private Employment Agencies (Amendment) Act 2017.

The past several years, particularly the post-2018 era: Malaysia’s first change of federal government at the 14th general election have seen notable developments that signal national impetus to reset migrant workers’ governance in the country. Several months after the formation of the newly elected government in 2018, an Independent Committee on Foreign Worker Management (ICFWM) was established with the primary mandate to conduct a thorough study on the recruitment of migrant workers. Later in 2019, 2020 and 2021, several more regulatory reforms were initiated such as, (i) amendment of the Employees’ Minimum Standards of Housing, Accommodations and Amenities Act (1990) in 2019; (ii) amendment of Employees’ Social Security (SOCSO) Act (1969) in 2019; (iii) amendment of Employment Act (1955) in 2022; (iv) renewal of third Malaysia’s National Action Plan on Trafficking in Persons (2021–2025); and (v) establishment of the first National Action Plan on Forced Labour (2021–2025)—all of which aimed at further strengthening workers’ employment protection and access to justice.

Concomitant with these notable developments, there have been growing public reporting and trade-related sanctions in recent years concerning migrant workers’ precariousness, including forced labour in Malaysia. These include the issuance of the U.S. Customs and Border Protection’s (CBP) withhold release orders (WRO) to several Malaysian companies between 2019 and 2021 (further see U.S. Customs and Border Protection, 2022). Existing studies indicate several potential systemic causes that persistently contribute towards workers’ precariousness. These include, for instance, the government’s failure to effectively implement labour and victims’ protection mechanisms in the country (see Wan Ismail et al., 2017; Wahab, 2019; Ormond & Nah, 2020). Other studies highlight that Malaysia’s efforts to strengthen the domestic legislations do not result in increased protection of labour trafficking victims (Devadason & Meng, 2014; Low, 2021a). This is because the effective protection of victims is dependent on a range of other factors, including effective access to legal representation and remedies (Wahab, 2019), and the ability of frontline enforcement personnel to exercise their duty and apply humanistic judgement when dealing with vulnerable victims (Wan Ismail et al., 2017).

Existing studies note that Malaysia has been able to innovate the recruitment process through digitalization initiatives, among others, the creation and implementation of the Foreign Workers Centralized Management System (FWCMS) (Low, 2021b). The intention was to seamlessly manage recruitment processes, but it has also led to a few drawbacks, including over-commercialization and monopolization by a select few private firms that dominate the industry. The digitalized recruitment process, nevertheless, is unable to address the complex nature of the recruitment industry both in Malaysia and sending countries such as Nepal. The industry continues to generate millions of dollars, but it is also featured by complicated profits and loss accounts due to the lack of transparency and accountability, and complex networks of enterprises and intermediaries in source and destination countries (Ajis et al., 2015; Wan Ismail et al., 2017). This complexity, compounded by privatization and monopolization of the recruitment industry, eventually increases the costs of recruitment, migrants’ dependency on private recruitment agencies and the risk of debt bondage.

## Drivers of the changing views of mobility

This section examines the employment conditions and life experiences among the Nepali workers in Malaysia during the pandemic, and how they are seen as the drivers of the changing views of mobility.

### Continuous practices of documents retention and restriction of freedom of movement

Retention of workers’ documents, including workers’ passports, is identified as one indicator of forced labour. Where retention of documents takes place, workers may face restrictions and administrative barriers to access them. In other common situations, migrant workers are strictly prohibited to access their passports until the time they are repatriated to their respective countries of origin. In Malaysia, legal safeguard to ensure workers keep their personal documents, including passports, is unclear. However, the latest administrative circular known as the ‘Employers Undertaking’, issued by the Malaysia’s Ministry of Human Resources (MOHR), has clearly prohibited employers to withhold migrant workers’ documents, including workers’ passports. Failing to adhere to this administrative requirement may cause employers prohibited to hire migrant workers in the future.

Despite such administrative sanction, withholding of migrant workers passports is still a common practice among many employers in Malaysia. In this study, we found that the vast majority of the Nepali worker informants (23 out of 28 Nepali worker informants) reported having their passports, at the time of the interview, were kept by their respective employers. Most workers cited that passport-keeping by employers is a common exercise, and in many cases, it is a requirement set by their respective employers. Workers claimed that passport-keeping was done immediately when they arrived in Malaysia, and they were only given a copy of their passports to avoid being arrested by the authorities. Nearly a dozen of Nepali informants added that they were given an immigration card (or *I-Card*, issued by Malaysia’s Immigration Department), along with a copy of their passports as a safeguard to prevent arbitrary arrest by the authorities. When asked under what circumstances they can access their original passport, some informants informed that they still could access the original passports if the workers travel across different states in Peninsular Malaysia—either for work or visiting friends. However, during the course of the pandemic, none of the workers reported having the opportunity of accessing their passports due to interstate travel restrictions.

Withholding of workers’ passports has been a growing concern among Nepali informants, especially during the COVID-19 pandemic. With the difficulty to physically meet and communicate with employers, workers had no chance of accessing their passports. Several Nepali informants further informed that their passports were about to expire in two to three months ahead (at the time of the interview), but employers were reluctant to respond and communicate with workers. These workers were made highly vulnerable to losing their legal status (i.e., become irregular migrant workers) for failing to renew their working passes on time. The continuous practices of withholding workers’ passports and challenges facing workers to access them at times of unprecedented situations such as the COVID-19 pandemic—have shaped the way they think about employers’ treatment of workers’ welfare, security, and safety in the country.

### Growing violations of labour rights

Interviews with Nepali workers also found a range of other labour rights violations occurred during the COVID-19 outbreak. These include seven Nepali workers in factories who had highlighted issues related to excessive hours of work and payment of wages that is not consistent with the signed contract of employment. Workers claimed that the factory's management instructed workers to work between 12 and 16 h a day. On some occasions, workers were asked to work such long hours for two weeks without a day off (i.e., continuously). When asked if workers were willing to work for such a long hour without a rest day, workers revealed a mixed response. Some workers expressed willingness to work long hours as long the overtime pay is commensurate with their hard work. Nevertheless, while these workers agreed to work for long hours, they were not paid according to their contract terms (i.e., being paid at a premium rate). Workers also highlighted that though they agreed to work overtime as instructed by the management, there was no procedure in place to seek workers’ consent.

In the meantime, some workers expressed unwillingness to work more than 12 h per day, and without a rest day in a week. These workers claimed that they need sufficient rest hours and time to socialize with their friends and communities, including during the pandemic period. One Nepali worker, 29-year-old, reported saying that there were nearly 10 occupational injury cases in 2021 alone in his workplace (i.e., a rubber glove factory) due to work fatigue. He claimed that these injured workers may face extreme tiredness and that reduced their functional capacity to work in the factory. He further claimed that most of these injury cases did not result in fair compensation to workers.

Interviews with Nepali community leaders in Malaysia highlighted other common violations of labour rights facing Nepali workers in Malaysia, especially during the COVID-19 pandemic. One of the Nepali community leaders, 41-year-old, claimed that he received several complaints from workers who were not given their salary for between six and 12 months in 2020 and 2021. Other grievances he received from his community members include salary deduction for the purpose of purchasing personal protective equipment (PPE) at workplaces, and COVID-19-related protective equipment such as hand sanitiser and masks. He further informed that it is the responsibility of the employers to provide these items to workers for free. He stressed that any deduction of salary for these purposes is deemed unlawful. Another Nepali community leader, 37-year-old, expressed concern over the growing complaints about the retrenchment of many Nepali workers in Malaysia during the COVID-19 pandemic. He informed that some of these Nepali workers were retrenched without prior notice (to workers). Without upfront notice, workers did not have enough time to find a new employment, causing them at higher risk of becoming irregular migrant workers: meaning losing their legal employment status and are at risk of arrest and deportation.

### Financial remittance is the last option

During different phases of MCOs in Malaysia, migrant workers were among the most affected by the reduced workforce in the business sector by 50 per cent or more, following the strict COVID-19 standards operating procedure (SOP) imposed by the government. This had severe implications for many Nepali workers, especially those that are paid as daily-wage earners and productivity-based workers (meaning: workers who are paid based on the amount of work or productivity they produce on a daily basis). Some Nepali workers working in the manufacturing, services and security sectors, informed that they were still allowed to work during MCOs but there was a drastic reduction in the number of hours and days they were allowed to work. This situation reduced the workers’ monthly income drastically. For those who were not permitted to work at all during the MCO, they had no income for several months up to one year.

Loss of income and the drastic reduction in monthly wages had serious financial consequences to Nepali workers. Given the high costs of living in Malaysia (e.g., meals, housing and telecommunication), some Nepali workers had to deprioritize financial remittance back to their origin country. Most Nepali worker informants highlighted that they were barely sending money back to their families in 2020. One of them, aged 33-year-old, specifically highlighted that he only remitted money back to his wife and five children in Nepal once in 2021. For him, this was an unusual practice, but he had to make this tough decision with the hope that he can survive and stay for a few more years to enable him to cover the recruitment cost he bore when he first migrated to Malaysia in 2018/2019.

Another Nepali worker aged 37-year-old agreed that the value of financial remittance has been significantly reduced during the MCOs period. However, he claimed that there have been growing digital platforms offering financial remittance services to Nepal. He added that easy access to internet connectivity has made it less troublesome for many Nepali workers to remit money back home, only by using their smartphones. This increased the frequency of financial remittances though the value (i.e., in US$) is getting lesser during the pandemic. When asked whether such remittance platforms were official and legit, he was unsure. However, he was quick to assure that the money he remitted in the past had been successfully received by the beneficiaries back home.

Financial remittance during the pandemic is certainly an important aspect for migrant workers’ families and dependents in Nepal, simply because they too were affected by the pandemic. However, Nepali worker informants in Malaysia expressed their worries concerning the reduced value of their remittance due to the significant loss of income in Malaysia. Rapid digitalization of remittance facilities, as claimed by a Nepali informant, helped in increasing the frequency of remittance but not the value (i.e., in US$). The reduced value of remittance during the pandemic eventually developed perceptions that labour mobility to Malaysia does not live up to their initial aspiration of mobility.

### During the COVID-19 outbreak, no one is hearing us!

Interviews with Nepali workers also found serious dissatisfaction with the way their grievances were managed by their respective employers, especially during the COVID-19 period. Workers alleged that the employers were not competent and honest in addressing their grievances. Grievances raised by the Nepali worker informants were allegedly not resolved in a way that benefited the workers.

The past few years have shown growing resources allocated to establish credible grievance mechanisms that enable workers to raise their grievances for solutions. These include external grievance channels established by Malaysia’s Ministry of Human Resources (MOHR) known as ‘*Working for Workers* (WfW)’ (accessible at https://www.workforworkers.com.my/sapn-portal/index), enabling workers in Malaysia to lodge their complaints and grievances through a smartphone application. Besides, there have been efforts by NGOs to introduce independent grievance platforms such as ‘*Just Good Work*’ (accessible at https://justgood.work/) for vulnerable migrant workers to report their grievances for immediate solution. As a common practice for other migrant workers, Nepali workers are also able to lodge their complaints and grievances to the Nepali Embassy in Kuala Lumpur, Malaysia for the necessary intervention.

Despite the various grievance mechanisms available to workers, Nepali worker informants reported that they were only aware of the operational grievance mechanism provided by their respective employers. When asked if they knew other external grievance mechanisms, none of the workers was able to indicate their knowledge about the ‘*Working for Workers*’ application (i.e., government-led grievance channel) and ‘*Just good Work*’ (i.e., NGO-led grievance channel). When asked if workers attempted to raise their grievances to external parties such as the embassy or NGOs, workers were clueless about the processes needed to raise their grievances. One Nepali worker, 29-year-old, raised that no one was actually keen to listen to their problems during COVID-19 period because everyone is busy saving their life.

### Where are the Nepali community leaders in Malaysia?

In Malaysia, there are Nepali community leaders who play an active role in helping and advocating the rights of Nepali workers. Most community leaders are connected to a range of formal institutions such as the Nepali Embassy in Malaysia and local Non-Governmental Organizations (NGOs). The Nepali community leaders also serve as the informal grievance channel, which then brings the issues raised to the attention of the employers and authorities for resolution. Interviews with Nepali community leaders confirmed that they receive direct suggestions, complaints and grievances from Nepali workers across different sectors and workplaces. In fact, the Nepali community leaders reported having representatives located in different states across Peninsular Malaysia where Nepali workers are present.

Interviews with Nepali workers however found that they barely raised their issues to community leaders though they knew some of them. When asked why, some Nepali workers claimed that the community leaders were also not competent in addressing their grievances. Nepali workers claimed that some Nepali community leaders requested upfront money if they were to be assisted. Other Nepali workers were clueless about the presence of Nepali community leaders.

A follow-up interview with some Nepali community leaders confirmed that they indeed requested upfront money from the workers to help them address the grievances. These community leaders stressed that the upfront money aimed at facilitating their travel and documentation—not a service fee. Besides, one of the community leaders, 44-year-old, claimed that the requested upfront money is not excessive though no particular amount was mentioned. Finding a resolution for any grievances raised by workers requires the community leaders to arrange several face-to-face meetings with the employers. In some cases, they had to bring the case to the attention of the Nepali Embassy in Kuala Lumpur. This requires travelling costs and resources, including the possibility of getting legal advisors and counsel to help bring the case to court.

Another Nepali community leader in Malaysia, 37-year-old, specifically highlighted that his community group is not registered and runs on a voluntary basis. To be able to manage grievances, they expect a small contribution from the workers. When asked if he received grievances from workers during the COVID-19 pandemic, he cited receiving grievances related to non-payment of wages, including non-payment of overtime hours, lack of compensation (in case of occupational injury) and employment retrenchment, especially during the COVID-19 outbreak.

### Nepali workers under outsourcing companies are the first to be ‘sacrificed’

Interviews with Nepali workers also found that there were cases where some Nepali workers were still hired and paid directly by the outsourcing companies, rather than direct hiring by their respective employers. These workers were mainly the workers, at the time of the interview, working in electronic manufacturing (factories) companies in Selangor and Penang. The Nepali workers claimed that as ‘indirect employees’ of the factories, they were vulnerable to a range of employment risks, especially during the course of the pandemic. One of which was the risk to be first retrenched in response to the reduced workforce by 50 per cent or more, following the strict COVID-19 SOP imposed by the government. Two Nepali workers informed that they were the first group of workers in their factory to be retrenched during the first phase of MCO back in March 2020. The two workers were unemployed and subsequently lost their income for nearly a year. However, they were later rehired in early 2021 by the same factory management through their outsourcing company.

A group of other Nepali workers who were also under an outsourcing company highlighted that as ‘indirect employees’ of a manufacturing factory in Selangor, they had no legitimate avenue to raise their concerns and grievances, especially during the pandemic period. When asked what their grievances were—some Nepali informants reported having issues concerning their wages being unfairly deducted. When they raised such grievances to their outsourcing company, they were instructed to raise them to factory management. When they raised it to factory management, they were in turn asked to raise it to the outsourcing companies. One of the Nepali worker informants, 32-year-old, said “… *we are like a ping-pong ball*”.

Interviews with Nepali community leaders confirmed that there are still workers hired by third-party outsourcing companies, particularly in the service and manufacturing sectors in Malaysia. A community leader, 43-year-old, claimed that though these ‘indirect Nepali workers’ are not hired directly by the manufacturing or service companies, the workers were often asked to adhere to the work rules set by the factory or service company management. These include the provision and wearing of PPEs, allocation of overtime work, and salary calculation. Salary calculation is among the common issues and misunderstandings that occur between workers and the management body. Besides, many ‘indirect Nepali workers’ reside in the accommodation provided by the factories (i.e., management) instead of the outsourcing companies. In this situation, workers often find it difficult to communicate or reach out to outsourcing companies.

### COVID-19, digitalization and the changing views of mobility

In this section, we explain the intersection between the changing views of mobility and the current discourse of migration aspiration and mobility model. Here, we focus on two specific themes, namely, digitalization and the COVID-19 pandemic, and their likely impacts on mobility in the Nepal–Malaysia migration corridor.

#### COVID-19, digitalization, and migration aspiration

With the technologies that are easily attainable to both Nepali workers in Malaysia and their relatives and friends in Nepal, remote communication becomes frequent. For some Nepali workers in Malaysia, it is routine for them to have a daily remote conversation over the phone with families and relatives back in Nepal. Besides, easy access to technological devices such as smartphones and laptops enables workers to get connected with families, relatives, and friends within Malaysia, and between Malaysia and Nepal. Social networks platforms such as *WhatsApp*, *WeChat* and *Facebook* are among the most common platforms used by many Nepali workers in Malaysia. Besides, Nepali workers in Malaysia use virtual social network applications as a platform to share information. During the pandemic, Nepali workers actively shared information on these social network platforms regarding such topics as current immigration regulations, job opportunities, COVID-19 SOPs and regulations, health-related news and public donation. A Nepali migrant worker in Malaysia, 35-year-old, said:

Internet cost is cheaper now. As long as you have a smartphone, you can talk to your family in Nepal. During the COVID-19 outbreak, since we were at home most of the time, I had WhatsApp call all day long with my children and friends in Nepal. Sometimes I complained about my salary getting lesser during the pandemic, and other Nepali friends in Malaysia who were even abandoned by their employers.

Virtual interviews with Nepali community leaders in Nepal highlighted that the Nepali migrant workers in Malaysia actively shared information virtually regarding passport retention and other forms of mistreatments (e.g., unpaid wages) during the pandemic. In fact, stories about Nepali workers who died in Malaysia during the pandemic were raised on several *Facebook* pages, eventually picked up by the local news in Nepal. This attracted attention from the Nepali politicians and NGOs in the country. One of the Nepali community leaders in Nepal, 39-year-old, highlighted that the Nepali migrant workers who died in Malaysia is not uncommon. These incidents happened even before the pandemic. However, recent issues about massive retrenchment involving thousands of Nepali workers in Malaysia, compounded with the great loss of income and drastic reduction of remittance to Nepal brought fresh and troubling perceptions about labour migration to Malaysia.

Virtual information sharing has made these stories and work experiences in Malaysia reached the aspiring Nepali migrants, politicians, and members of the public—relatively easier, faster, and uncensored. This may shape or build negative perceptions about labour mobility to Malaysia. One Nepali community leader in Nepal, 48-year-old, informed that while many Nepali individuals in Nepal are looking forward to working abroad in the post-COVID-19 period, Malaysia is becoming less attractive to many aspiring Nepali workers. The rapid flow of information due to digitalization is likely to make up one’s mind about labour migration into Malaysia. He said:

Good salary, overtime work and work benefits are always the key motivations for many aspiring Nepali workers to work abroad, especially Malaysia. Once they migrated, they are expected to remit money to their family back here in Nepal. So, if Malaysia is unlikely to offer a good salary, well I think many Nepali individuals will go somewhere else. But if Malaysia continues to offer competitive income, Malaysia is still the place of choice.

It becomes apparent now that the social construction of migration is shaped, influenced, and inspired by virtual information sharing between Nepali workers abroad, and their relatives and friends in Nepal. Here, digitalization and technologies play a seemingly important role in building perceptions and aspirations, and eventually influencing the decision-making process among Nepali households in Nepal.

#### COVID-19 pandemic, and migration aspiration and ability

The previous section has explained the precarious working and living conditions among Nepali workers in Malaysia, particularly during the pandemic. Nepali workers in Malaysia were continuously deprived of their right to earn a stable and fair income, enjoy their freedom of movement, and effective access to justice and remedy. Such precarious working and living conditions eventually serve as the drivers of the changing views of mobility. It is important to note that these precarious working and living conditions are not new, as explained in the earlier section. However, these precarious conditions have positioned workers even more physically and economically vulnerable (e.g., loss of income and reduced value of remittance).

Nepal is one of the world’s top largest recipient countries receiving remittances from Nepali workers abroad. In 2016, the country received a total of US$ 8.1 billion in remittances from millions of Nepali workers abroad (Sah, 2019). Remittances become the major contributing factor to increasing household income in the country and contributing about 30 per cent to Nepal’s Gross Domestic Product (GDP). Interestingly, as mentioned before, nearly 50 per cent of Nepali households rely on financial help from relatives abroad (Kunwar, 2020). This indicates the high level of reliance among Nepali households, including families and dependents of Nepali workers abroad on monetary remittances.

Against this backdrop, we present several possibilities of the likely impacts of the aggravated employment precariousness among Nepali migrant workers in Malaysia in shaping or influencing the social construction of migration in Nepal. First, the aggravated precarious conditions among the Nepali workers in Malaysia is likely to produce more ‘voluntary non-migrants’—a term used by Carling (2002) to refer to individuals who prefer to stay (or not to migrate) because of a belief that non-migration is preferable to migration. Our logic is simple. The aggravated precarious conditions during the pandemic signal a strong message that labour migration to Malaysia is not only physically unsafe but financially disadvantaged (e.g., due to drastic reduction in monetary wage or income).

Secondly, the significant reduction in monetary remittances received by families and dependents in Nepal has eventually reduced the household income and savings in the country. Similarly, like other countries affected by the pandemic, the employment and financial sectors in Nepal were severely affected. Access to capital and loans was getting tougher and more costly (i.e., high-interest rates). Given the financial pressure and limited access to financial services (e.g., loans), it is likely that more aspiring Nepali workers in the country are financially incapable to migrate for work. This points out the likely growing number of ‘involuntary non-migrants’ in the country—a term also used by Carling (2002) to refer to individuals who aspire to migrate but lack the ability to do so.

Third, the financial constraints to support labour migration, intensified by the growing mobility restrictions in many source countries such as Malaysia and the Gulf countries are likely to drive more Nepali individuals who are aspired to migrate to decide using the irregular migration facilities. Globally, patterns of irregular migration are dynamic and complex, but commonly involve unlawful international border crossing activities (Spencer & Triandafyllidou, 2022). This may be case for many aspiring workers in Nepal. However, given the geographical barriers (i.e., long-distance travel from Nepal to Malaysia), we do not anticipate this group of workers to migrate into Malaysia’s territories, but Nepal’s neighbouring countries such as India.

#### What does it imply to future labour migration in the Nepal–Malaysia migration corridor?

Notwithstanding the three possibilities presented before, the past five years have already shown a steady reduction in the number of Nepali workers migrating to Malaysia (Seng Guan & Anita, 2022), yet Nepali workers are still the top three migrant worker groups hired in such major economic sectors as manufacturing and services. On the side of Nepal, as mentioned before, it had also been severely impacted by the spread of COVID-19 with a nationwide lockdown measure imposed to contain the virus between March and July 2021. Many Nepali individuals lost their jobs, while others shut down their informal and small enterprises. In fact, a serious food stock issue had been reported (Sharma et al. 2021). Interviews with Nepali community leaders in Nepal highlighted that many Nepali individuals are now looking forward to migrating to help rebuild their life, including using irregular migration services to migrate to such countries as India.

With the current development in Nepal, the aspiration to migrate among Nepali individuals is high. The Nepali workers’ employment and life experiences in Malaysia during the COVID-19 outbreak, supported by digitalization—are obviously shaping the aspiring Nepali workers’ perceptions of labour migration to Malaysia. However, it does not necessarily suggest that labour migration in the Nepal–Malaysia migration corridor is going to be less significant, especially during the post-COVID-19. At the time of writing this article, Malaysia has lifted the entry restrictions for five countries: India, Sri Lanka, Pakistan, Bangladesh and Nepal, enabling migrant workers from these countries to enter Malaysia for work (Kalra, 2021). The decision to lift the entry restriction was a strategic policy response to address critical labour shortage facing many Malaysian employers in the country.

## Conclusion

Given the strong demand and supply market sentiments in Malaysia and Nepal, labour mobility in the corridor is still highly relevant, and it is expected to continue especially when COVID-19-related mobility restrictions are fully lifted by the end of 2022. However, the scale of such labour mobility is difficult to ascertain. The migration aspiration and ability model offers an insightful and practical theoretical framing to better understand and reflect international labour mobility in the Nepal–Malaysia migration corridor. To reiterate, it is not the intention of this study to forecast the trend of labour migration. Rather, to examine narratives of the changing views of mobility and the likely impacts of the pandemic on labour mobility in the Nepal–Malaysia migration corridor.

In this study, we contribute to the ongoing debate about the aspiration and ability model by highlighting two themes for further scholarly discourse. First, the impacts of digitalization on the social construction of migration especially in the source country. The digitalization process is not a new phenomenon (see Alencar et al., 2018) but it evolved more prominently during the pandemic. Second, the impacts of COVID-19 on migrant workers in destination countries such as Malaysia, and how the pandemic drives the new narratives that shape views of mobility among the aspiring workers in source countries such as Nepal.

Finally, we echo Carling and Schewel (2018) that the nature of migration aspiration is an empirical matter. It is built, shaped, and influenced by the social construction of migration that exists not only in source and destination countries respectively but between them. Similarly, migration ability evolves evermore challenging, compounded not only by the limited access to capital and financial services but restrictive mobility regulations. It is difficult to ascertain how labour mobility would appear in the post-COVID-19 period. It all depends on how governments, especially the Nepali and Malaysian Governments respond to, and reorient their respective labour mobility policies and interventions.

## Data Availability

The study is based on qualitative interview materials which were collected on the basis that they remain anonymous and confidential.
